# Bone Substitute Fabrication Based on Dissolution-Precipitation Reactions

**DOI:** 10.3390/ma3021138

**Published:** 2010-02-10

**Authors:** Kunio Ishikawa

**Affiliations:** Department of Biomaterials, Faculty of Dental Science, Kyushu University, 3-1-1 Maidashi, Higashi-ku, Fukuoka 812-8582 Japan; E-Mail: ishikawa@dent.kyushu-u.ac.jp; Tel.: +81-92-642-6344; Fax: +81-92-642-6348

**Keywords:** apatite, phase transformation, dissolution-precipitation, carbonate apatite, low-crystallinity, precursor

## Abstract

Although block- or granular-type sintered hydroxyapatite are known to show excellent tissue responses and good osteoconductivity, apatite powder elicits inflammatory response. For the fabrication of hydroxyapatite block or granules, sintering is commonly employed. However, the inorganic component of bone and tooth is not high crystalline hydroxyapatite but low crystalline B-type carbonate apatite. Unfortunately, carbonate apatite powder cannot be sintered due to its instability at high temperature. Another method to fabricate apatite block and/or granule is through phase transformation based on dissolution-precipitation reactions using a precursor phase. This reaction basically is the same as a setting and hardening reaction of calcium sulfate or plaster. In this paper, apatite block fabrication methods by phase transformation based on dissolution-precipitation reactions will be discussed, with a focus on the similarity of the setting and hardening reaction of calcium sulfate.

## 1. Introduction

**Figure 1:**
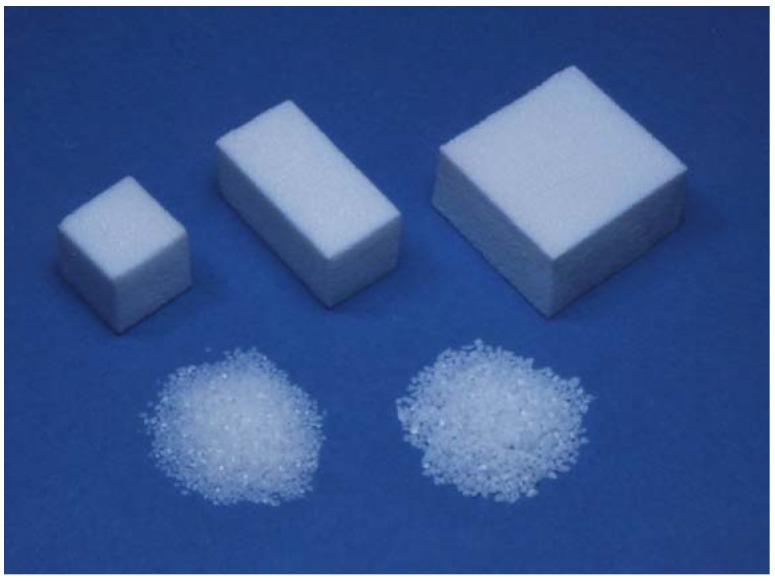
Example of sintered HAp used for the artificial bone substitutes.

Although sintering is commonly used for the fabrication of ceramic, it results in high crystallinity with less specific surface area. In the case of health-related bioceramics, sometimes bioceramic materials with low crystallinity and larger specific surface area are desired to have higher tissue compatibility and/or other biological functions. Also, some of the bioceramics are unstable at the high temperature required for the sintering process. One of the examples is bone apatite. Apatite is a mineral name that has the chemical structure of M_10_Z_6_X_2,_ where M can be Ca^2+^, Cd^2+^, Sr^2+^, Ba^2+^, or Na^+^; Z can be PO_4_^3-^, CO_3_^2-^, SO_4_^2-^, or SiO_4_^2-^; and X can be OH^-^, F^-^, Cl^-^, or CO_3_^2-^ [1]. As can be seen, many elements can be incorporated in apatitic structure. Table 1 summarizes the example of bone apatite [1]. As can be seen in this Table, bone apatite is calcium phosphate apatite containing significant amount of carbonate and many trace elements. The chemical structure of bone apatite is Ca_10-a_Mg_b_Na_c_K_d_(PO_4_)_6-e_(CO_3_) _f_(OH)_2-g_Cl_h_F_i_ but could be stated as Ca_10-a_(PO_4_)_6-b_(CO_3_)_c_(OH)_2-__d_ if trace elements in which the content is less than 1 wt % are eliminated. Therefore, bone apatite should be called carbonate apatite (CO_3_Ap). Unfortunately, bone apatite is not stable at the high temperatures required for the sintering process due to the significant amount of carbonate that thermally decomposes to other calcium phosphate. In the 1970’s, Prof. Aoki found that carbonate-free stoichiometric hydroxyapatite (HAp; Ca_10_(PO_4_)_6_(OH)_2_) can be sintered, and that it showed excellent tissue response and good osteoconductivity, *i.e.*, the existing bone is bonded with sintered HAp when implanted adjacent to the bone. It was also found that the osteoconductivity of HAp is higher when sintered at lower temperature [2,3,4,5,6]. Introduction of sintered HAp allowed fruitful results to the patients, since no osteoconductive materials were available up until the invention of sintered HAp. Also, invention of sintered HAp may be the beginning of recent bioceramic research. Many sintered HAp products are available for clinical use in block and granular form as shown in Figure 1.

**Table 1:** Hard tissue components of the human adult. Weight % except for Ca/P molar ratio [1].

	Enamel	Dentine	Bone
Ca^2+^	36.5	35.1	34.8
PO_4_ as P	17.7	16.9	15.2
Ca/P molar ratio	1.63	1.61	1.71
			
Na^+^	0.5	0.6	0.9
Mg^2+^	0.44	1.23	0.72
K^+^	0.08	0.05	0.03
CO_3_^2-^	3.5	5.6	7.4
F^-^	0.01	0.06	0.03
Cl^-^	0.30	0.01	0.13
P_2_O_7_^4-^	0.022	0.10	0.07
Total Inorganic	97.0	70.0	65.0
Absorbed H_2_O	1.5	10.0	10.0

Although sintered HAp was a breakthrough for biomaterials aimed for the reconstruction of bone defects, it also has shortcomings for some clinical cases. A key drawback is its stability in the implanted site. It should be noted that the stability and relatively high mechanical strength of sintered HAp are advantageous for some clinical cases. In other words, requirements for biomaterials differ depending on the clinical case. In some clinical cases, bone substitute is desired to replace new bone since artificial HAp cannot replace biological roles of bone such as hematogenesis. Also, sintered HAp shows too high Young Modulus, which result in stress shielding to the surrounding bone and causes bone resorption. In contrast to sintered HAp, grafted bone can be replaced by new bone. Therefore, autograft, or bone taken from the patient, is still regarded as a golden standard for the reconstruction of bone defects even though its use has been decreasing continuously. Autograft has serious drawbacks, including invasion to healthy sites for bone collection, limitation of the collectable bone in volume and in shape, functional decrease and infection risk at the bone collection site, and increased medical cost for additional surgery. These shortcomings can be solved if artificial bone replacement can be developed.

Phase transformation based on dissolution-precipitation reactions is one of the very useful methods for the fabrication of bioceramics similar to bone apatite. In other words, apatite with low crystallinity, high specific surface area, and containing carbonate ion can be fabricated using phase transformation of unstable precursors based on dissolution-precipitation reactions. In this review, phase transformation based on dissolution-precipitation reactions is explained using the setting and hardening reaction of calcium sulfate and apatite cement, followed by its application for apatite block fabrication.

## 2. Setting and Hardening of Calcium Sulfate by Phase Transformation Based on Dissolution-Precipitation Reactions

**Figure 2:**
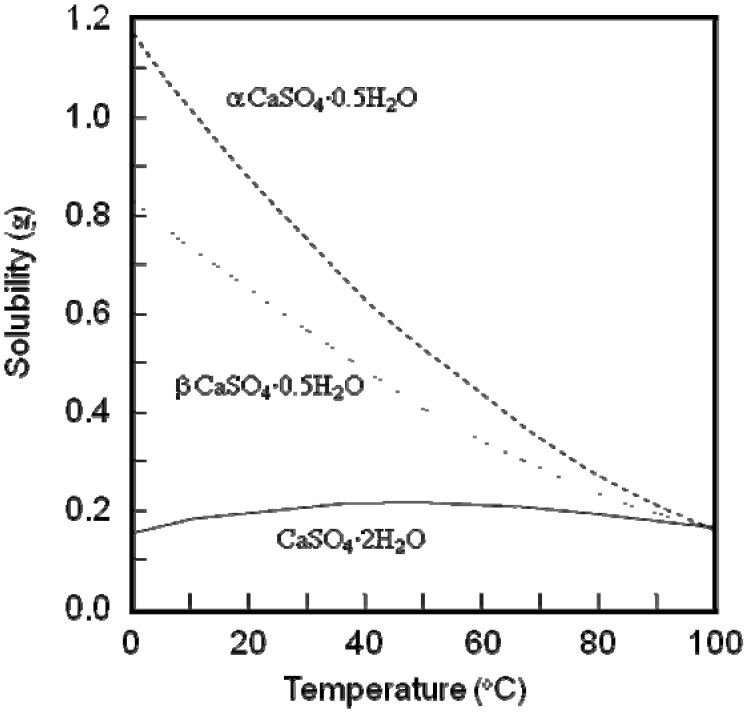
Solubility of α and β calcium sulfate hemihydrate and calcium sulfate dihydrate as a function of temperature [13].

Phase transformation based on dissolution-precipitation reactions is well known as the setting and hardening reaction of calcium sulfate hemihydrates known as plaster [11,12,13,14]. Calcium sulfate hemihydrates (CaSO_4_^.^0.5H_2_O) set and harden to form calcium sulfate dihydrate (CaSO_4_^.^2H_2_O) since calcium sulfate dihydrate is more stable thermodynamically in the presence of water than calcium sulfate hemihydrate, and the precipitated calcium sulfate dihydrate crystals interlock with each other. In other words, there are two requirements for the fabrication of stable phase by the phase transformation based on dissolution-precipitation reaction. One requirement is the moderate instability, *i.e.*, moderate solubility of the precursor when compared to the final block product. Due to the instability of the precursor, the precursor phase is dissolved in aqueous solution and supplies ions that are required for the precipitation of the final product. Figure 2 shows the solubility of calcium sulfate hemihydrates and dihydrate as a function of temperature.

For example, the solubility of α-calcium sulfate hemihydrates is approximately 0.9g at 20 °C. When α-calcium sulfate hemihydrates are mixed with water, Ca^2+^ and SO_4_^2-^ which correspond to 0.9 g, CaSO_4_^.^0.5H_2_O is supplied into 100 g water as shown in equation (1). If α CaSO_4_^.^0.5H_2_O is the only calcium sulfate, it will reach equilibrium and no further reaction will occur. However, calcium sulfate dihydrate has also equilibrium with Ca^2+^ and SO_4_^2-^ as shown in equation (2), and its solubility is approximately 0.2g at 20 °C. Due to difference in solubility of the two calcium sulfate salts, CaSO_4_^.^0.5H_2_O sets and hardens to form CaSO_4_^.^2H_2_O.

αCaSO_4_^.^0.5H_2_O ⇄ Ca^2+^ + SO_4_^2-^ + 0.5H_2_O (1)

CaSO_4_^.^2H_2_O ⇄ Ca^2+^ + SO_4_^2-^ + 2H_2_O (2)

CaSO_4_^.^0.5H_2_O + 0.5H_2_O → CaSO_4_^.^2H_2_O (3)

**Figure 3:**
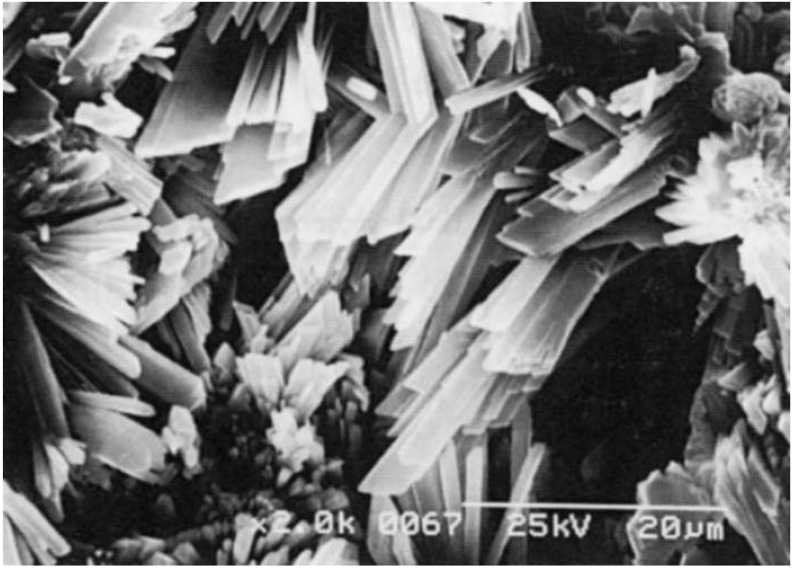
Typical scanning electron microscopic image of hardened calcium sulfate. Needle-like calcium sulfate dihydrate crystals interlock with each other to form hardened calcium sulfate [13].

Exposure of CaSO_4_^.^0.5H_2_O to water results in the dissolution of CaSO_4_^.^0.5H_2_O basically to reach its equilibrium and supplies Ca^2+^ and SO_4_^2-^, which correspond to 0.9g CaSO_4_^.^0.5H_2_O. However, the aqueous solution is supersaturated with respect to CaSO_4_^.^2H_2_O since its solubility is 0.2g. Therefore, Ca^2+^ and SO_4_^2-^, which correspond to approximately 0.7g CaSO_4,_ will be precipitated as CaSO_4_^.^2H_2_O. The precipitation of Ca^2+^ and SO_4_^2-^ as CaSO_4_^.^2H_2_O crystals results in undersaturation with respect to CaSO_4_^.^0.5H_2_O, leading to the dissolution of CaSO_4_^.^0.5H_2_O, which results in the supersaturation of aqueous solution with respect to CaSO_4_^.^2H_2_O. As a result of consecutive dissolution-precipitation reactions, formed CaSO_4_^.^2H_2_O crystals grow and interlock with each other to set and harden as shown in Figure 3.

**Figure 4:**
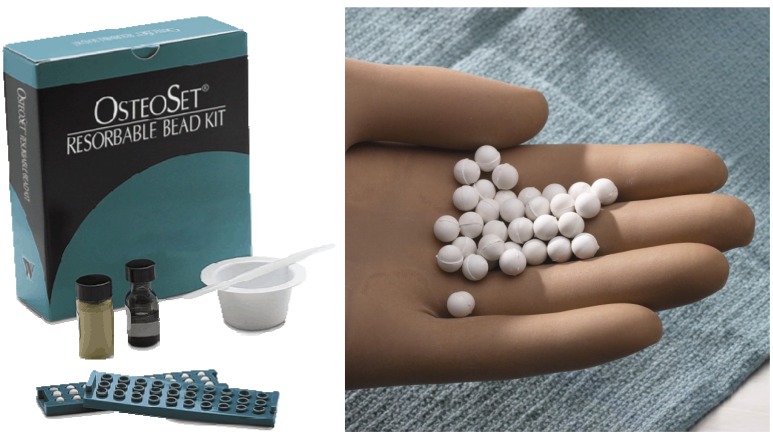
Example of calcium sulfate clinically used for the reconstruction of bone defects (Photo courtesy of Wright Medical Technology).

**Figure 5:**
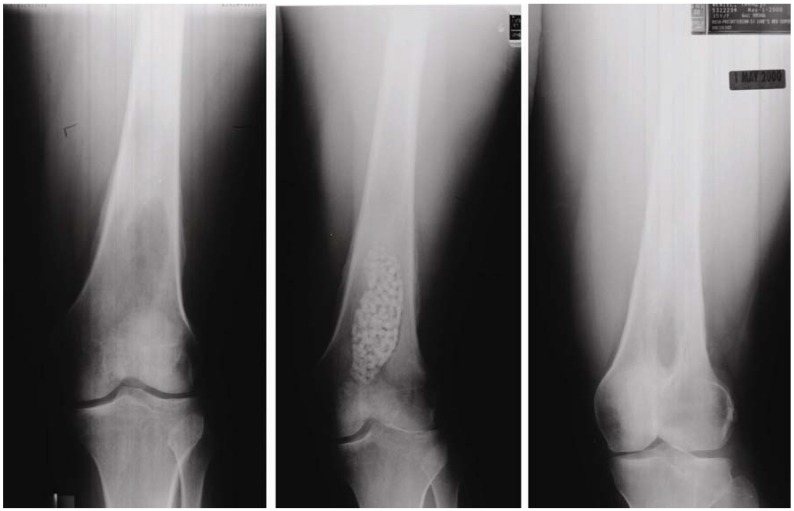
Radiographic assessment of OsteoSet(R) resorbable beads filled into the bone void of a 40-year-old female resulting from osteomyelitis, taken at: preoperation (left), postoperation (middle), and follow-up (right). (Photo courtesy of Wright Medical Technology).

Calcium sulfate sets and harden by phase transformation reaction based on dissolution-precipitation reaction and has the longest history for its use as an artificial bone substitute [12,15,16], for an example, seeFigure 4 and Figure 5. Key properties of calcium sulfate are its rapid and complete resorption with minimal inflammatory response [17,18,19,20]. This is due to the relatively large solubility of calcium sulfate dihydrate. It has also been found that granular hydroxyapatite-like calcium phosphate mineral is deposited on the surface of resorbing calcium sulfate [21,22]. Of course, hydroxyapatite-like calcium phosphate mineral is thought to be formed by the phase transformation reaction based on the dissolution-precipitation reaction. Calcium sulfate dihydrate dissolves and supplies Ca^2+^ and SO_4_^2-^ to the body fluid since body fluid is undersaturated with respect to calcium sulfate dihydrate. However, once the concentration of Ca^2+^ is elevated, based on the dissolution of calcium sulfate, the body fluid will be supersaturated with respect to apatite, and thus, apatite crystal is formed. The formed hydroxyapatite-like calcium phosphate mineral may act as an osteoconductive “trellis”. Therefore, osteoconductivity of calcium sulfate is also caused by the superficial phase transformation of calcium sulfate based on dissolution-precipitation reaction.

## 3. Setting and Hardening of Apatite Cements by Phase Transformation Based on Dissolution-Precipitation Reactions

Apatite cement, which sets and harden like gypsum and forms apatite as a final product was initially invented by Professors Monma and Kanazawa in 1976 [23]. They reported that α-tricalcium phosphate (α-TCP; α- Ca_3_(PO_4_)_2_) set to form calcium-deficient apatite with a Ca/P molar ratio of 1.5 when α-TCP was hydrated in water at 60–100 °C and with a pH between 8.1 and 11.4 [24]. The α-TCP and Ca-deficient apatite are in equilibrium with Ca^2+^ and PO_4_^3-^ in aqueous solution as shown in equations (4) and (5).

3αCa_3_(PO_4_)_2_ ⇄ 3Ca^2+^ + 2PO_4_^3^ (4)

Ca_9_(HPO_4_)(PO_4_)_5_(OH) ⇄ 9Ca^2+^ + 6PO_4_^3-^ + H_2_O (5)

3αCa_3_(PO_4_)_2_ + H_2_O → Ca_9_(HPO_4_)(PO_4_)_5_(OH) (6)

**Figure 6:**
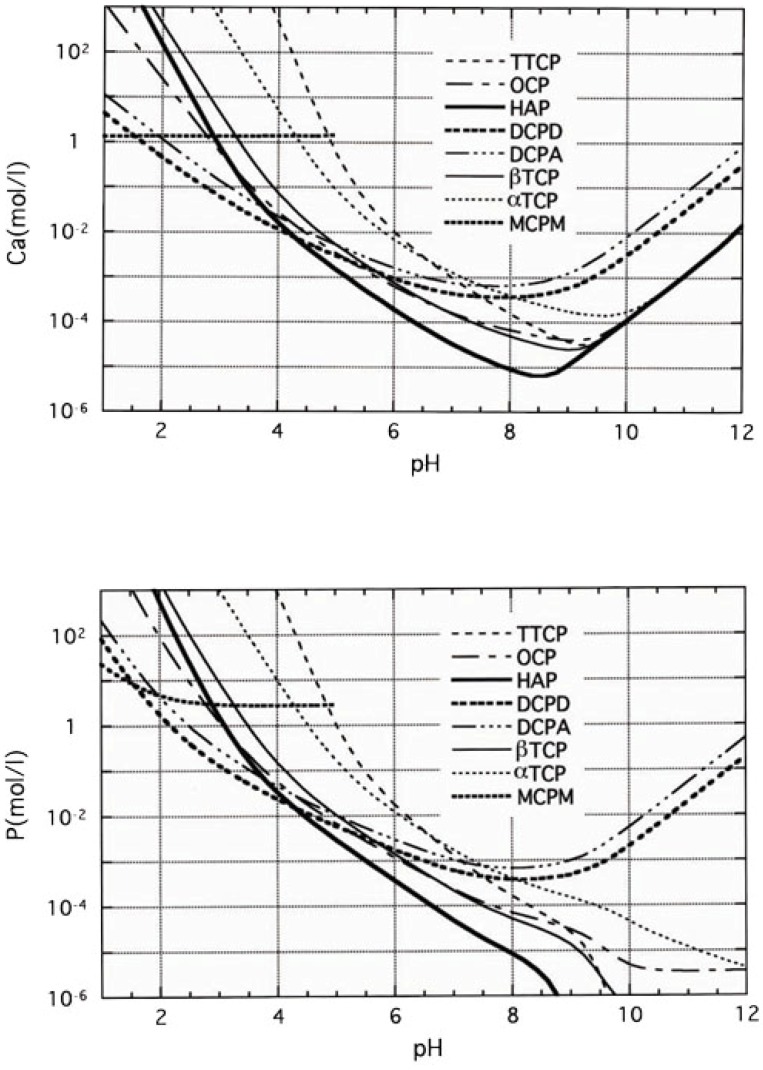
Solubility of Ca and P in various calcium phosphate compounds plotted against the pH calculated from their solubility products [13,14].

When comparing α-TCP and Ca-deficient apatite, α-TCP is unstable and Ca-deficient apatite is the stable phase thermodynamically at physiological conditions as shown in Figure 6. Therefore, α-TCP supplies Ca^2+^ and PO_4_^3-^ when exposed to aqueous solution. However, the resulting aqueous solution is supersaturated with respect to Ca-deficient apatite, leading the precipitation of Ca-deficient apatite crystals. As a result of consecutive dissolution-precipitation reactions, precipitated Ca-deficient apatite crystals interlock with each other to set and harden.

The key drawback of apatite cement consisting of α-TCP is its relatively hard setting condition. The α-TCP-type apatite cement was shown to need 60–100 °C and 24 h to set [20]. This condition is too hard and too long for clinical use. Therefore, dicarboxylic acid such as succinic acid or citric acid was employed in the liquid phase to acquire an initial setting reaction [24,25]. Dicarboxylic acids are known to chelate calcium-containing powder and thus an initial setting reaction of α-TCP-type apatite cement can be achieved by the chelating reaction. At the same time, dicarboxylic acid inhibits the apatite formation. Also, dicarboxylic acid is thought to reduce dissolution reactions by covering the surface of α-TCP. Therefore, both dissolution and precipitation reactions are inhibited by the introduction of dicarboxylic acid. However, the amount of dicarboxylic acid is limited when compared to the amount of α-TCP, and thus the dissolution-precipitation continues even in the presence of dicarboxylic acid and results in the hardening reaction.

The reaction becomes more complicated in the case of apatite cement consisting of an equimolar mixture of tetracalcium phosphate (TTCP: Ca_4_(PO_4_)_2_O), and dicalcium phosphate anhydrous (DCPA: CaHPO_4_), which was invented by Drs Brown and Chow in 1986 [26,27]. TTCP and DCPA dissolves faster when compared to α-TCP. Therefore, TTCP-DCPA type apatite cement sets within approximately 30–60 min at physiological temperature and forms apatitic product when mixed with aqueous solution. Although two calcium phosphate powders are used for this apatite cement, the basic reaction is the same as with the other cement, *i.e.*, phase transformation based on dissolution-precipitation reactions. TTCP and DCPA equilibrates with Ca^2+^ and PO_4_^3-^ as shown in equations (7) and (8).

Ca_4_(PO_4_)_2_O + H_2_O → 4Ca^2+^ + 2PO_4_^3-^ + 2OH^-^ (7)

CaHPO_4_ ⇄ Ca^2+^ + H^+^ + PO_4_^3-^ (8)

Ca_10_(PO_4_)_6_(OH)_2_ ⇄ 10Ca^2+^ + 6PO_4_^3-^ + 2OH^-^ (9)

2Ca_4_(PO_4_)_2_O + 2CaHPO_4_ → Ca_10_(PO_4_)_6_(OH)_2_ (10)

**Figure 7:**
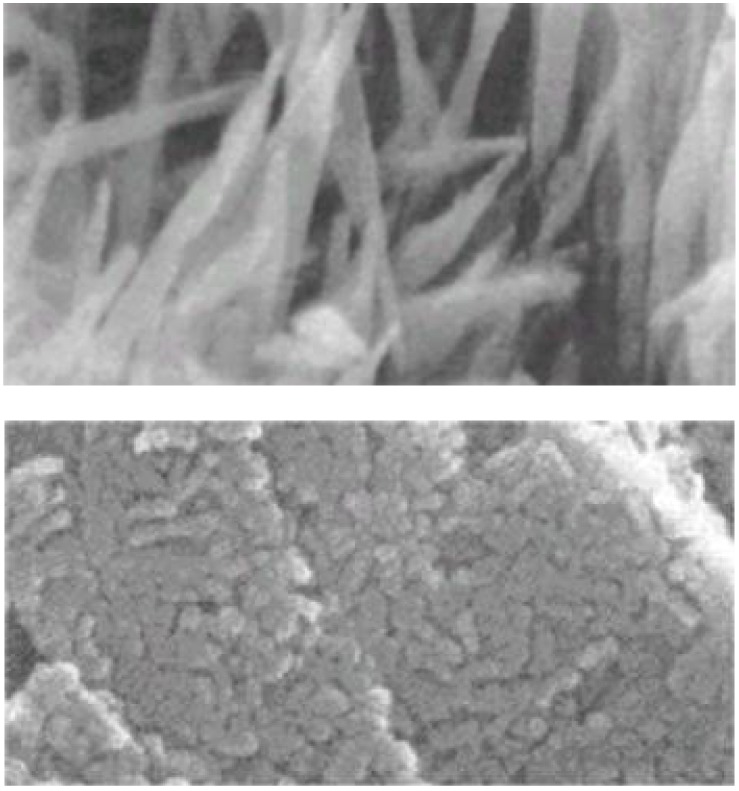
Typical scanning electron microscopic image of apatite cement consisting of TTCP and DCPA. Upper image = surface; lower image = interior [13,14].

When compared with TTCP, DCPA and apatite, apatite is the unstable phase. Actually, apatite is the most stable phase thermodynamically at physiological condition as shown in Figure 6. Apatite also has equilibrium with Ca^2+^ and PO_4_^3-^ as shown in equation (9). When, TTCP and DCPA are exposed to aqueous solution, they dissolve and supply Ca^2+^ and PO_4_^3-^. However, the resultant aqueous solution is supersaturated with respect to apatite, leading to the precipitation of apatite crystals. Precipitation of the apatite crystals decreases the concentration of Ca^2+^ and PO_4_^3-^, which leads to further dissolution of TTCP and DCPA. The dissolution-precipitation basically continues until all TTCP and DCPA is consumed for the precipitation of apatite crystals. The precipitated apatite crystals interlock with each other to set and harden. Since the mechanism of the setting and hardening reaction of apatite cement is exactly the same as that of gypsum, hardened apatite cement also shows similar morphology, as shown in Figure 7.

**Figure 8:**
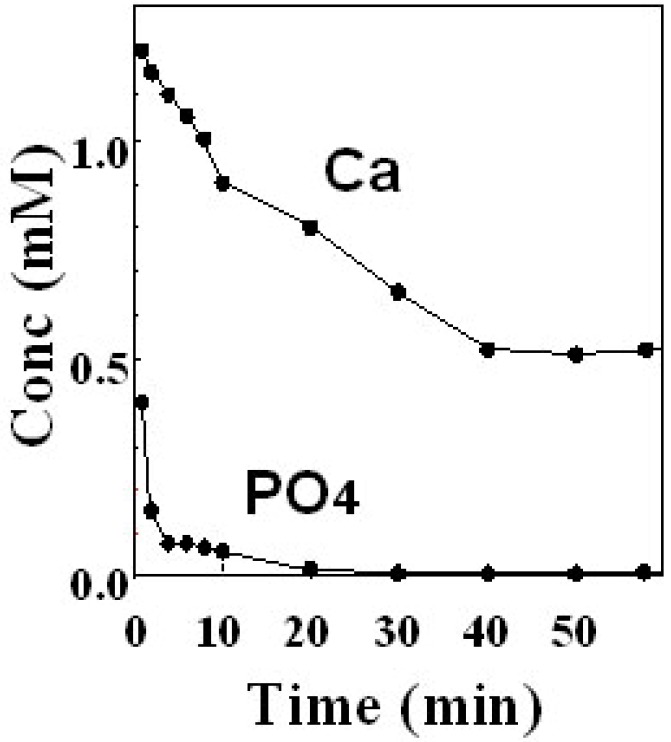
Example of the calcium and phosphate ions concentration in the suspension of apatite cement containing TTCP and DCPA. Calcium ion kept some concentration whereas phosphate concentration became less than detection limit over time [13].

The key difference between TTCP-DCPA type apatite cement and α-TCP type apatite cement or calcium sulfate is that the former consist of two calcium phosphate salts whereas the powder phase of the latter two consists of single salts. When two salts are contained in the powder phase, the regulation of powder size becomes much more important than when compared with single phase cement. Table 2 summarizes the example of the effects of particle size on the mechanical strength of the final products. A mixture containing small TTCP and large DCPA did not set in 24 hours. Mechanical strength increased with diameter ratio of TTCP to DCPA, to reach a maximum value when the mixture contained large TTCP and small DCPA. This was caused by the different dissolution rate of TTCP and DCPA. In other words, TTCP dissolves more rapidly than DCPA if the particle sizes are the same. However, both TTCP and DCPA need to be dissolved simultaneously for the apatite crystals to grow. In order to adjust the dissolution rate, combination of large TTCP and small DCPA is desired. However, DCPA with approximately 1 μm is the smallest powder that can be obtained using mill grinding method. Mixture of 1 μm diameter DCPA powder with 10 μm diameter TTCP means DCPA powder has a 100 times larger surface area when compared to TTCP. However, this combination is still not enough for the simultaneous supply of Ca^2+^ and PO_4_^3-^ for the apatite crystal growth. As shown in Figure 8, ion analysis of TTCP-DCPA mixture revealed a shortage of PO_4_^3-^.

**Table 2:** Effects of particle size on the compressive strength of calcium phosphate cement consisting of tetracalcium phosphate and dicalcium phosphate anhydrous [13].

Average particle diameter (μm)		Ratio of the average particle diameter of TTCP/DCP	Compressive strength (MPa)
TTCP	DCPA		
1.6	11.9	0.13	0 (no setting)
12.4	11.9	1.04	7.1 ± 1.0
1.6	0.9	1.78	21.8 ± 4.4
12.4	0.9	13.78	51.0 ± 4.5

 Again, both Ca^2+^ and PO_4_^3-^ need to be supplied for the precipitation of apatite crystals. When PO_4_^3-^-containing aqueous solution is used as liquid phase of TTCP-DCPA type apatite cement, setting time was shortened significantly from 30–60 min to 5 min [28,29], see Table 3. The significant shortening of the setting time is caused by the supply of PO_4_^3-^ and results in the immediate increase in the degree of supersaturation with respect to apatite. The apatite cement using phosphate salts such as Na_2_HPO_4_ was named fast-setting apatite cement to distinguish it from the conventional apatite cement that employs water as liquid phase [28,25]. However, the use of phosphate salt has became common at the present time, and thus use of the term "conventional" or "fast-setting" may not be proper at present.

**Table 3:** Effects of liquid phase on the setting time of calcium phosphate cement consisting of tetracalcium phosphate and dicalcium phosphate anhydrous [13,28].

Liquid	Concentration (mol/L)	Setting time (min)
Distilled water	-	30–60
Na_1.8_H_1.2_PO_4_^*^	0.2	5
Na_1.8_H_1.2_PO_4_	0.6	5
Na_1.8_H_1.2_PO_4_	1.0	5
K_1.8_H_1.2_PO_4_	0.2	5
K_1.8_H_1.2_PO_4_	0.6	5

## 4. Phase Transformation Based on Dissolution-Precipitation for the Fabrication of Carbonate Apatite Block

The setting and hardening reactions of calcium sulfate or the apatite cements are phase transformations based on dissolution-precipitation reactions, and the interlocking of the precipitated crystals as stated above. In the case of both calcium sulfate and apatite cements, the unstable phase was the powder. Of course, phase transformation based on dissolution-precipitation reactions also occurs when the unstable phase is the granular or the block form. For the fabrication of a CO_3_Ap block, the unstable phase or precursor block should contain, at least, one of the elements of CO_3_Ap, and the precursor should show proper solubility. For example, CaCl_2_ cannot be a precursor since it dissolves too quickly. On the other hand, CaCO_3_ contains both Ca^2+^ and CO_3_^2-^ required for the fabrication of CO_3_Ap, and has moderate solubility. Since phase transformation based on dissolution-precipitation occurs only at the surface of the precursor, an interconnected porous structure is one of the requirements for the precursor. Also, the precursor should not be washed out even when the precursor is immersed in liquid.

Calcium carbonate blocks with an interconnected porous structure can be fabricated by exposing calcium hydroxide to carbon dioxide as shown in Equation (11).

Ca(OH)_2_ + CO_2_ → CaCO_3_ + H_2_O (11)

CaCO_3_ ⇄ Ca^2+^ + CO_3_^2^ (12)

Ca_10-a_(PO_4_)_6-b_(CO_3_)_c_(OH)_2-__d__⇄_ (10-a)Ca^2+^ + (6-b)PO_4_^3-^ + c CO_3_^2-^ + (2-d)OH^-^ (13)

(10-a)CaCO_3_+(6-b)Na_2_HPO_4_ + (2-d)H_2_O → Ca_10-a_(PO_4_)_6-b_(CO_3_)_c_(OH) _2-__d_ + (10-a-c)CO_3_^2-^ + (12-2b)Na^+^ + (2-d)H^+^ (14)

The calcium carbonate fabricated by this method was calcite, one of the polymorphs of calcium carbonate. It had an interconnected microporous structure and would not be washed out even when the block was immersed in aqueous solution [30,31].

When a calcite block was immersed in aqueous solution, calcite dissolved and supplied Ca^2+^ and CO_3_^2-^ to the aqueous solution as shown in equation (12). If the aqueous solution contains no other ions, the solution will be at equilibrium with respect to calcite and no further reaction would occur. On the contrary, if the aqueous solution contained PO_4_^3-^, the solution is also at equilibrium with carbonate apatite as shown in equation (13). Since the solubility of carbonate apatite is much smaller compared to calcite, the solution would be supersaturated with respect to CO_3_Ap. Therefore, Ca^2+^ and CO_3_^2-^ supplied by the dissolution of calcite is precipitated with PO_4_^3-^ as CO_3_Ap as shown in equation (14). The precipitation of Ca^2+^ and CO_3_^2-^ as CO_3_Ap results in the undersaturation of the aqueous solution with respect to calcite. Therefore, calcite further dissolves and supplies Ca^2+^ and CO_3_^2-^ in the aqueous solution. The reaction continues until all calcite or Na_2_HPO_4_ is consumed or exposure of calcite to aqueous solution is prevented by the precipitated CO_3_Ap or due to other reasons. The precipitated carbonate apatite was found to be B-type, in which replacement of CO_3_ occurs at the PO_4_ site or B site instead of the OH site or A-site. B-type carbonate apatite is known as the type of carbonate apatite found in bone.

**Figure 9:**
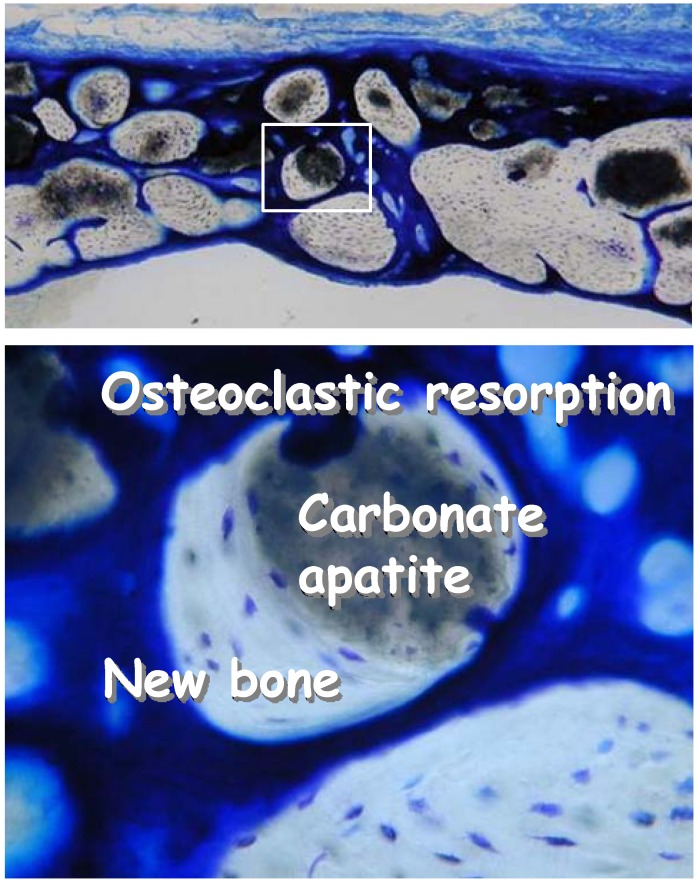
Histological pictures of carbonate apatite 24 weeks after implantation in a bone defect made to the cranial bone of rats. After 24 weeks, most of the carbonate apatite granules were replaced by new bone. Osteoclastic resorption can be seen along with new bone when carbonate apatite was not replaced.

When CO_3_Ap granules fabricated in this phase transformation based on dissolution-precipitation reaction was implanted in defective bone, CO_3_Ap was found to be replaced with bone similar to autograft as shown in Figure 9.

A calcite block needs to be fabricated from calcium hydroxide, since calcium carbonate compact would be washed out when immersed in aqueous solution. On the contrary, calcium sulfate hemihydrate sets and hardens as calcium sulfate dihydrate. Hardened calcium sulfate would not be washed out and has a moderate solubility of 0.2 g/100 mL (see Figure 2). Therefore, calcium sulfate can also be a precursor for the fabrication of CO_3_Ap or HAp [33,34].

**Figure 10:**
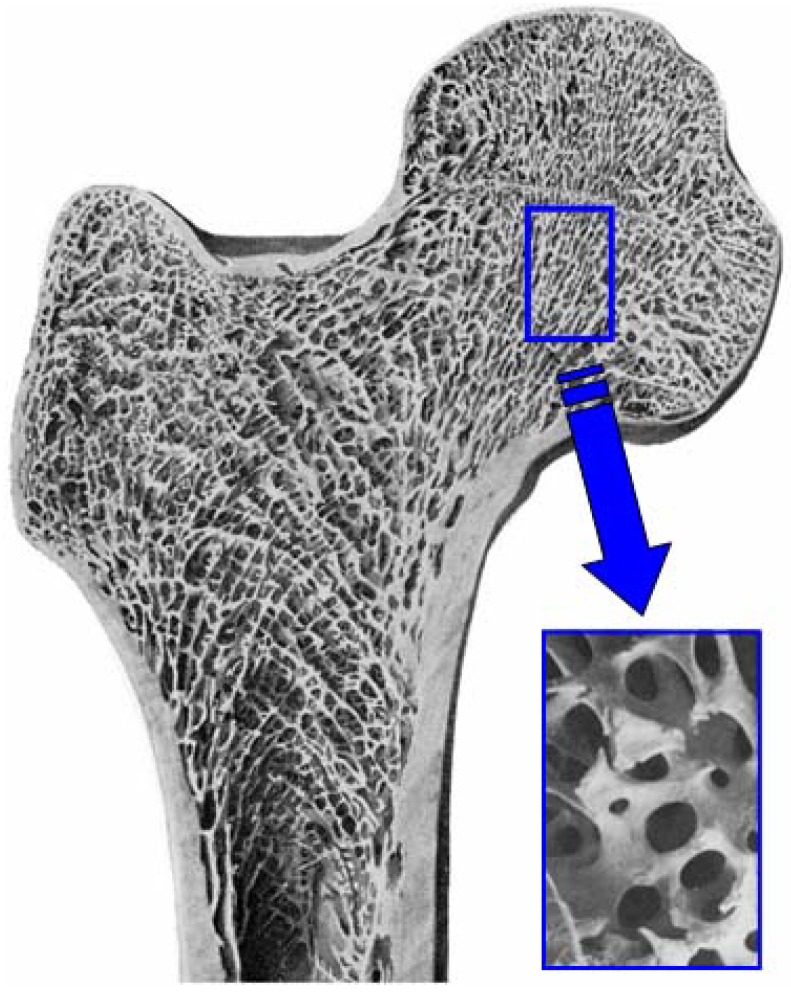
Example of cancellous bone. Cancellous bone has fully interconnected porous structure.

**Figure 11:**
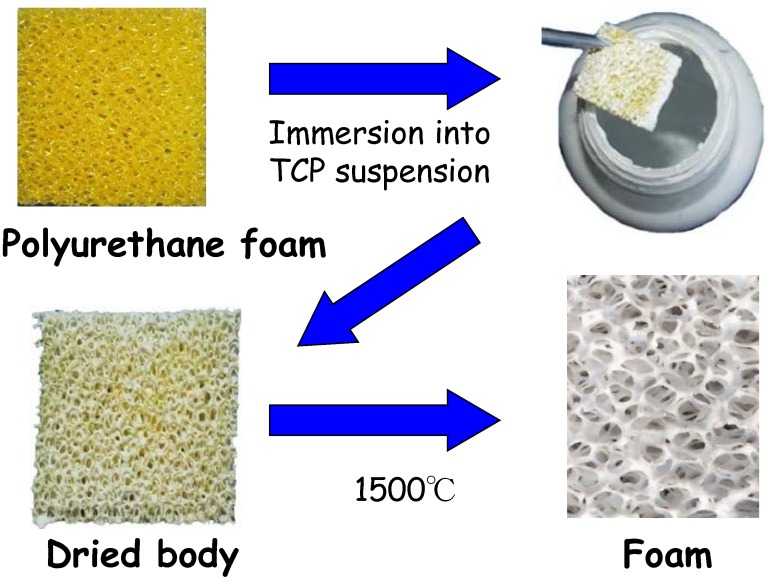
Procedure for the fabrication of α-TCP foam.

Both calcium carbonate and calcium sulfate are not stable at high temperature. Hence, the precursor needs to be fabricated at a low temperature. In contrast, α-TCP is the stable phase at high temperature. Therefore, the α-TCP precursor can be fabricated by different methods including the sintering process [35]. For example, the α-TCP precursor is useful for the fabrication of CO_3_Ap foam. As shown in Figure 10, cancellous bone has a fully interconnected porous structure, which is ideal for the penetration of tissues and cells. This structure can also be seen in polyurethane foam. The α-TCP foam can be fabricated as shown in Figure 11. First, polyurethane foam is immersed into TCP slurry to allow the coating of the polyurethane foam surface with TCP. Heating the TCP-coated polyurethane foam to a high temperature results in sintering of TCP and burning out of the polyurethane foam. The TCP foam thus prepared has almost the same structure as polyurethane foam or cancellous bone. The α-TCP foam precursor transforms to apatite or carbonate apatite by phase transformation based on dissolution-precipitation reactions when exposed to distilled water or carbonate salt containing solution, respectively [36,37,38].

## 5. Granular Apatite Cement

**Figure 12:**
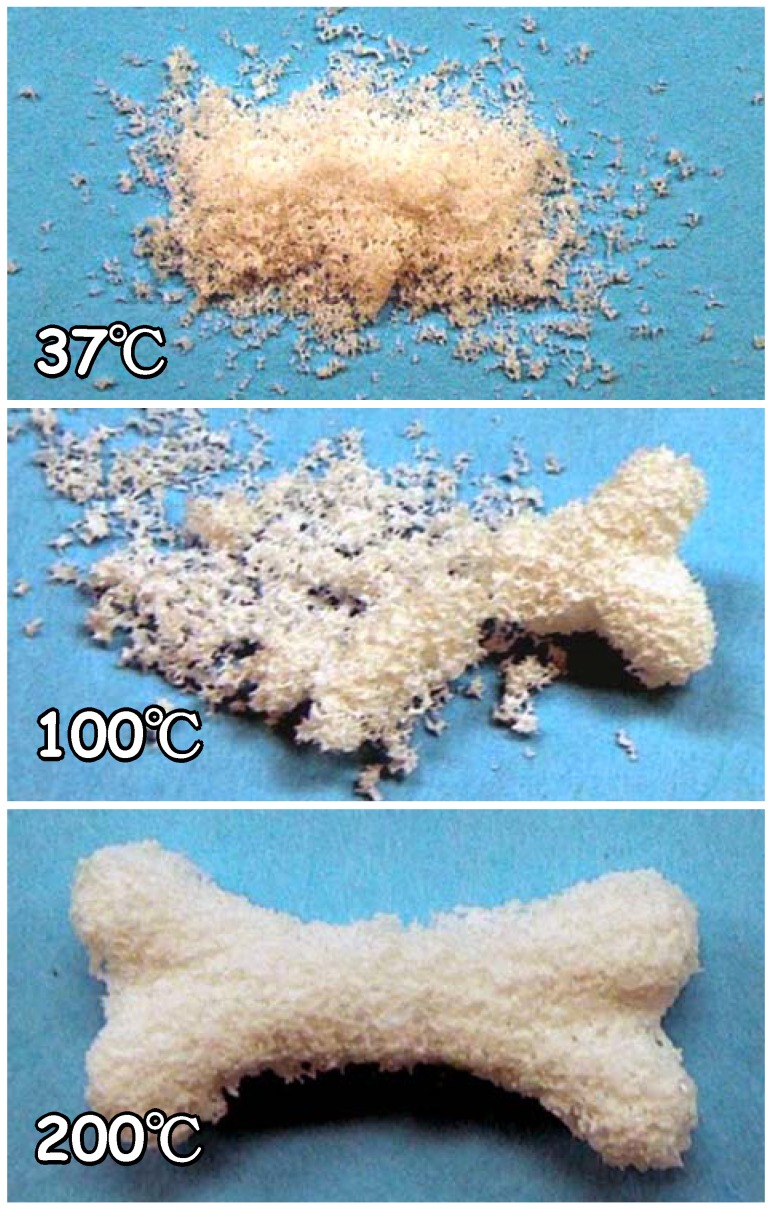
Photographs of specimens of α-TCP foam granules after immersion at 37 °C (top) and after hydrothermal treatment at 100 °C (middle) and 200 °C (bottom) for 24 h [39].

Calcium sulfate hemihydrate and apatite cements set and harden by phase transformation based on dissolution-precipitation reactions and interlocking of precipitated crystals as stated previously. The precursor phase of calcium sulfate and apatite cements are powders. On the other hand, apatite block or carbonate apatite block can be fabricated by phase transformation based on dissolution-precipitation reactions. If so, block type apatite and carbonate apatite should be able to be fabricated based on the setting and hardening reaction since precipitated crystals formed by the dissolution-precipitation reaction should have the ability to interlock with each other. It was found that α-TCP granules made by crushing α-TCP foam, set and hardens to form apatite foam as shown in Figure 12 [39].

The key difference between the apatite cement and apatite granule cement is its surface area. As stated above, one of the key factors influencing the apatite cement is the regulation of powder size. In the case of granular apatite cement, regulation of the granular size aiming for increased surface area is almost impossible. Also, area that can interlock with each other is limited. To enhance the interlocking, a higher temperature is employed. In other words, crystal growth can be expected at higher temperatures. For example, the condition needed for the fabrication of the fully interconnected porous structure shown in Figure 12 was 200 °C when the reaction time was 24 hours. At 37 °C, almost no setting reaction was observed. At 100 °C, phase transformation based on dissolution-precipitation could not be completed in 24 hours. Also, the length of the precipitate crystals were shorter when compared to the specimen prepared at 200 °C. Although interlocking of the precipitate crystals is much more difficult when compared to powder precursor, the granular precursor could set and harden by phase transformation based on dissolution-precipitation reactions.

## 6. Future of Bioceramics Made with Phase Transformation Reactions Based on Dissolution-Precipitation Reactions

Phase transformation based on dissolution-precipitation reactions is expected to play very important roles in the fabrication of high functional bioceramics. Invention of the self-setting apatite cements is held as one of the breakthroughs in reconstruction for bone defects. It can be used for drug delivery devices, since hardened materials shows low crystallinity and high specific surface area, as well as a setting and hardening reaction process without generating heat that could damage the drug or the protein. CO_3_Ap block prepared by the phase transformation based on dissolution-precipitation reactions is replaced by bone through bone remodeling processes. To increase the benefit of bioceramics made with phase transformation reactions based on dissolution-precipitation reactions, further understanding of the reaction and improvement of the mechanical and/or biological function of bioceramics may be required.
